# Comparing surgical techniques: ThuLEP and transurethral BPEP for prostate over 80 grams. Intraoperative and postoperative results. A prospective randomized trial

**DOI:** 10.1080/20905998.2024.2395594

**Published:** 2024-09-11

**Authors:** Samer Morsy, Mahmoud Elfeky, Sherif Abdel-Rahman, Hesham Torad, Ahmed Rammah, Mina Safwat

**Affiliations:** a Urology Department, Cairo University Hospitals, Cairo, Egypt; b Urology Department, Misr University for Science and Technology, 6th of October City, Egypt

**Keywords:** BPH, enucleation, ThuLEP, BPEP, comparing

## Abstract

**Background and purpose:**

Though TURP remains the primary treatment for BPH, advancements in energy and technology have introduced innovative transurethral surgical options. In this study, we assessed and compared the effectiveness and safety of using thulium laser and bipolar for endoscopic enucleation of prostate exceeding 80 g.

**Patients and methods:**

Between January 2022 and July 2023, this study enrolled patients with LUTS due to BPH and a prostate size of ≥80 g. Group A underwent the BPEP procedure using a 26 Fr continuous flow resectoscope with plasma kinetic system enucleation loops, while Group B underwent the ThuLEP procedure using a 120–200 W Revolix DUO® Thulium laser. Data collection included prostate size, PSA levels, enucleation and morcellation time, and postoperative IPSS and IIEF-5 scores at one, 3, 6, and 12 months.

**Results:**

A total of 108 patients, divided into Group A (BPEP) and Group B (ThuLEP), completed a 12-month follow-up. The mean age for group A was 67.72 ± 7.02 compared to group B which was 62.33 ± 5.86. While Group A compared to group B had higher mean enucleation (75.22 ± 10.55 vs. 67 ± 12.18) and total operative times (117.22 ± 17.76 vs.90.5 ± 18.29) (*p* = 0.037 & <0.001 respectively), no significant differences were observed in resected tissue weight, blood transfusion, and morcellation time. The ThuLEP group exhibited a shorter mean catheter period 2.94 ± 0.94 d compared to BPEP 3.33 ± 0.91 d and shorter mean hospital stay period of 1.94 ± 0.54 compared to2.11 ± 0.32, though not statistically significant. Postoperative outcomes, including IPSS, Qmax, PVRU, and IIEF-5 at 1, 3, 6, and 12 months, showed no differences between the groups.

**Conclusion:**

ThuLEP shows better perioperative parameters in comparison to BPEP. Nevertheless, there are no notable differences in functional results and complications between the two techniques.

## Introduction

The primary treatment considered most effective for moderate-to-severe lower urinary tract symptoms (LUTS) caused by benign prostatic hyperplasia (BPH) is transurethral resection of the prostate (TURP). Nevertheless, in cases involving enlarged prostates, TURP remains associated with significant adverse effects, such as bleeding and transurethral resection syndrome (TURS) [1]. Recent advancements in energy and technology have given rise to several innovative transurethral surgical approaches, including minimally invasive surgical techniques (MIST). Consequently, this has diversified the range of options available for the treatment of BPH [2]. BPEP (Bipolar plasma enucleation of the prostate) is considered a superior alternative to TURP, surpassing both bipolar and monopolar TURP in terms of morbidity and the length of hospitalization [3,4]. With comparable prostate tissue removal to open prostatectomy and good surgical efficiency, it stands as a viable surgical choice for patients with significant BPH [5,6]. The technique of thulium laser enucleation of the prostate (ThuLEP) was initially introduced in 2010 by Herrmann and his colleagues [7]. During postoperative follow-up, ThuLEP exhibited equivalent safety concerning local complications and demonstrated similar effectiveness in terms of measures such as Qmax, International Prostate Symptom Score (IPSS), post-void residual (PVR), and quality of life (QoL). Nonetheless, it presented several advantages, including a decreased drop in hemoglobin levels, shorter durations of hospitalization, and reduced catheterization times [8–10].

Studies comparing BPEP and ThuLEP as regard perioperative data are still scarce. So in this study, we assessed and compared the effectiveness and safety of both methods.

## Patient and methods

From January 2022 to July 2023, patients were recruited from the outpatient clinics of Cairo University Hospitals and Souad Kafafi University Hospital, Misr University for Science and Technology. Patients with LUTS due to BPH-induced infravesical obstruction, an International Prostate Symptom Score (IPSS) ≥8, a peak urine flow rate (Qmax) of less than 15 ml/sec, and a prostate size of 80 g were enrolled in this study. Patients who were using anticoagulant or antiplatelet drugs, diagnosed with neurogenic bladder, urethral stricture, bladder stones, prostate cancer, or had previously undergone prostate or urethral surgery been excluded from the study.

An informed written consent was obtained from each patient before the start of the study. Patients were informed before surgery by all potential risks of the surgery. The study protocol was approved by the Research Ethics Committee of Urology Department, Faculty of Medicine, Cairo University. Code: MS-366-2022.

### Sample size and randomization

Based on the previously published data, the sample size was calculated according to mean enucleation time for ThuLEP [11] 70.5(58-87.3) min and BPEP [12] 56(23-75) minutes and the difference between them which was around 15 min Using student t-test with an 80% power and 5% α‐error level, the calculated sample size was 47 patients in each group. For the expected dropout during follow-up, we increased the sample size by 15% (54 patients in each group). Calculations were done using the MedCalc software’s power and sample size programme. A statistician randomized the eligible patients using block randomization and sealed envelopes. The participants were blinded to the assigned type of intervention at time of surgery. Patients were assigned into two groups. Group A represents the BPEP procedure, whereas group B represents the ThuLEP procedure.

### Data collection

Each patient underwent assessment through a medical history review, physical examination, and standard laboratory tests, encompassing urine analysis, urine culture, serum creatinine, complete blood count (CBC), coagulation profile, prostate-specific antigen (PSA), and electrolytes. The evaluation also included an assessment of IPSS, Qmax, and IIEF5. Ultrasound (US) was used to assess post void residual urine (PVR), as well as the kidneys and bladder. Trans rectal ultrasonography (TRUS) was used to measure prostate volume. When indicated, prostate biopsies were conducted.

### Intervention

The procedures were conducted under spinal/epidural anesthesia. A proficient surgeon with experience in performing over 50 cases per year of thulium or bipolar plasma kinetic enucleation carried out each procedure.

In group A, BPEP procedure was performed using a 26 Fr continuous flow resectoscope equipped with the plasma kinetic system’s enucleation loops (KARL STORZ HF Generator AUTOCON® III 400 and HERRMANN bipolar vapo-enucleation electrode) (KARL STORZ SE & Co. KG. In Tuttlingen, Germany). Power settings were 160 W for cutting and 80 for coagulation. In group B, the ThuLEP procedure was carried out using 120–200 W Revolix DUO® Thulium laser unit (Lisa laser, Katlenburg-Lindau, Germany) with a 550 μm RigiFib from Lisa Laser in Katlenburg-Lindau, Germany. In both techniques, a 26 Fr constant flow resectoscope was used with physiologic saline irrigation. Power settings were 60 W for cutting and 40 W for coagulation. Storz morcellator performed the mutilation (Karl Storz GmbH & Co., Tuttlingen, Germany).

The enucleation technique was the same in both groups, either two lobe or three lobe technique [13] but with early release of prostate apex to prevent traction on striated sphincter. Two incisions were made deep into the plane of the surgical capsule at the five and seven o’clock positions of the bladder neck, bringing them to the level proximal to the verumontanum. Beginning with the union of the two ends of the incisions at the level of the verumontanum, enucleation of the median lobe was initiated by bluntly pushing the tissue with the sheath of the resectoscope along the surgical capsule towards the 6-o’clock direction of the bladder neck. To enucleate the lobes, the resectoscope was moved to the 12-o’clock position, which opened the plane for enucleation of the upper portion of the lateral lobes. The lateral plane was developed to the verumontanum at the apex of the lobes during the enucleation phase, which involved blunt lifting in the direction of the bladder neck. Physiologic saline was used all throughout the procedures.

After adequate hemostasis, lobes pushed into the bladder were morcellated into tiny chips. Continuous irrigation was applied using a three-way 22-Fr silicone urethral catheter until the hematuria was resolved. When the catheter drainage remained clear after irrigation was stopped, the catheter was withdrawn. And before discharge, the PVR was assessed.

The primary outcome of the study was to assess the mean enucleation time for both techniques. Secondary outcome was to assess other perioperative parameters such as mean morcellation time, mean total operative time, and weight of resected prostatic tissues removed. Perioperative complications according to the Clavien–Dindo classification (Hb drop) and mean hospital stays and catheterization period. IPSS, IIEF, Qmax, and PVR at 1, 3, 6 and 12 months were assessed during follow-up in outpatients’ clinic.

### Statistical analysis

The statistical analysis was conducted using SPSS v26 (IBM Inc., Chicago, IL, USA). Mean and standard deviation (SD) were used to present quantitative variables, and a comparison between the two groups was performed using the unpaired Student’s t-test. Qualitative variables were presented as frequency and percentage (%), and analysis was carried out using the Chi-square test or Fisher’s exact test when applicable. A two-tailed *p* value <0.05 was deemed statistically significant.

## Results

**Figure 1.**
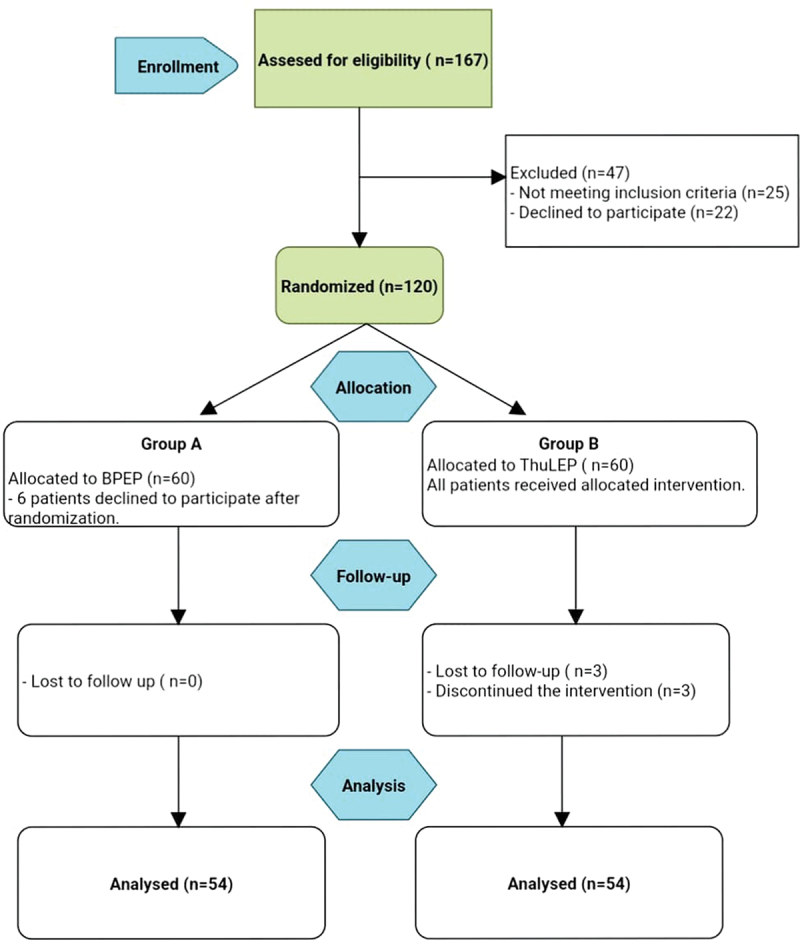
Consort diagram (participants flow diagram).

One hundred and eight patients altogether completed the study Figure 1, which included a 12-month follow-up period (54 patients in each group). The mean age for group A was 67.72 ± 7.02 compared to group B which was 62.33 ± 5.86 (*p* = 0.017). Urine retention (acute and chronic) was the main presentation for group A 54.4% and group B in 53.8% of the patients, followed by recurrent hematuria in (3.7% vs.1.8%) and finally, failed medical treatment in 38.9% of group A compared to group B 44.4%. All preoperative data including IPPS, IIE-F scores, Qmax, PVR, serum PSA and Hb were enrolled in Table 1.

**Table 1.** Preoperative assessment of studied groups.

		Group A (*n* = 54)	Group B (*n* = 54)	P value
IPSS score	Mean ± SD	22.33 ± 7.13	25.58 ± 2.07	0.138
IIEF-5 score	Mean ± SD	15.39 ± 2.25	15.11 ± 2.05	0.702
Preoperative Hb	Mean ± SD	13.3 ± 1.43	12.71 ± 0.75	0.130
Preoperative Na	Mean ± SD	140.1 ± 3.2 mmol/l	142.3 ± 2.4 mmol/l	0.901
Total PSA (ng/ml)	Mean ± SD	3.19 ± 1.61	4.19 ± 1.4	0.04*
Prostate volume (g)	Mean ± SD	101 ± 15.13	106.67 ± 19.04	0.330
PVR (ml)	Mean ± SD	133.39 ± 18.69	131.08 ± 18.81	0.744
Q_max_ (ml/s)	Mean ± SD	7.54 ± 2.31	6.83 ± 1.86	0.384

As regard perioperative data shown in Table 2, the mean enucleation time and mean total operative time were significantly higher in Group A (BPEP) (75.22 ± 10.55 & 117.22 ± 17.76), respectively, than Group B (ThuLEP) (67 ± 12.18 & 90.5 ± 18.29) (*p* = 0.037 & <0.001 respectively). The ThuLEP group showed a shorter catheter period and shorter hospital stay than BPEP but with no statistically significant difference. No patients were needed for 2nd look in Group A and Group B.

**Table 2.** Perioperative data.

Variable		Group A (*n* = 54)	Group B (*n* = 54)	P value
Enucleation time (min)	Mean ± SD	75.22 ± 10.55	67 ± 12.18	0.037*
Morcellation time (min)	Mean ± SD	26.5 ± 4.64	25.72 ± 5.14	0.637
Total operation time (min)	Mean ± SD	117.22 ± 17.76	90.5 ± 18.29	<0.001*
Weight of resected prostatic tissue (g)	Mean ± SD	102.72 ± 12.34	112.22 ± 18.67	0.081
Intraoperative blood transfusion	Yes No	2 (3.70%) 52 (96.29%)	1 (1.85%) 53 (98.15%)	0.546
Hemoglobin drop (g/dl)	Mean ± SD	1.84 ± 0.61	1.42 ± 0.69	0.058
Postoperative Na	Mean ± SD	138.5 ± 2.3 mmol/l	140.6 ± 2.7 mmol/l	0.832
Catheterization period (day)	Mean ± SD	3.33 ± 0.91	2.94 ± 0.94	0.215
Hospital stays (day)	Mean ± SD	2.11 ± 0.32	1.94 ± 0.54	0.269

As regard perioperative complications according to the Clavien–Dindo classification shown in Table 3, no statistically significant difference between both groups was documented. Capsular perforation occurred in one (1.85%) patient in group A and one (1.85%) patient in group B patient, minor bladder injury from morcellator occurred in 1 (1.85%) patient in group A and 1 patient in group B. No sub-trigonal dissection. Two patients in group A and one patient in group B received intraoperative blood transfusion due to low Hb level (<11.5 g./dl) before surgery. Stress urinary incontinence (SUI) in three patients in group A (5.56%) and four patients in group B (7.4%) with no statistically significant difference (*p* = 0.942) and improved within 3 months. Irritative urinary symptoms lasting longer than a week were treated with anticholinergic medications were used to treat them, and they disappeared after 1 month. One case (1.85%) in BPEP developed bladder neck contracture 3 months postoperatively and treated with bladder neck resection. No reported urethral stricture or secondary bleeding was found in the present study.

**Table 3.** Complications.

Grade	Group (A)	Group (B)	P value
Grade 1			
Capsule perforation	1(1.85%)	1(1.85%)	1
Retention of urine	0	0	1
Morcellation injury	1(1.85%)	1(1.85%)	0.942
Transient SUI	3 (5.6%)	4 (7.4%)	0.132
Irritative symptoms	4 (7.4%)	2 (3.7%)	
Grade 2:			0.235
UTI	1 (1.8%)	2 (3.7%)	
Blood transfusion	0	0	
Grade 3:		0	0.821
Bladder neck contracture	1(1.8%)		
Total Complications	11 (20.3%)	10 (18.5%)	1

Postoperative functional outcomes parameters results including IPSS, Qmax, PVR and IIEF-5 are shown in Table 4.

**Table 4.** Postoperative functional outcome at 1, 3, 6 and 12 months.

			Group A (*n* = 54)	Group B (*n* = 54)	P value
IPSS score	1 month	Mean ± SD	10.33 ± 2.3	9 ± 1.85	0.064
	3 months	Mean ± SD	8.39 ± 1.65	7.11 ± 2.32	0.066
	6 months	Mean ± SD	7.21 ± 1.21	6.13 ± 1.67	0.084
	12 months	Mean ± SD	5.83 ± 1.25	4.94 ± 1.47	0.059
IIEF-5 score	3 months	Mean ± SD	13.8 ± 2.1	14.1 ± 1.8	0.231
	6 months	Mean ± SD	14.8 ± 3.1	14.7 ± 2	0.381
	12 months	Mean ± SD	14.83 ± 1.76	14.39 ± 1.72	0.448
PVR (ml)	1 month	Mean ± SD	37.5 ± 19.42	35.83 ± 9.34	0.745
	3 months	Mean ± SD	17.61 ± 13.66	22.33 ± 5.75	0.185
	6 months	Mean ± SD	20.61 ± 16.62	21.36 ± 9.23	0.09
	12 months	Mean ± SD	12 ± 8.93	15.56 ± 4.3	0.137
Qmax (ml/s)	1 month	Mean ± SD	26.72 ± 5.24	25.83 ± 4.81	0.599
	3 months	Mean ± SD	27.83 ± 4.78	29 ± 3.31	0.400
	6 months	Mean ± SD	26.39 ± 6.6	27 ± 4.5	0.532
	12 months	Mean ± SD	30.39 ± 3.48	30.78 ± 4.85	0.784
PSA (ng/dl)	3 months	Mean ± SD	2.9 ± 1.2	2.5 ± 1.4	0.421
	6 months	Mean ± SD	1.8 ± 0.7	1.6 ± 0.8	0.782
	12 months	Mean ± SD	1.5 ± 0.8	1.6 ± 0.8	0.804
Prostate volume(ml)	12 months	Mean ± SD	20 ± 7.75 10 – 29	21 ± 6 13 – 30	0.352

## Discussion

Bladder outlet obstruction (BOO) frequently occurs as a complication of benign prostatic hyperplasia (BPH). Three key ideas from transurethral surgery serve as a starting point for the core principles of endoscopic BPH treatment. Vaporisation, enucleation, and ablation [14]. While TURP remains the predominant choice for treating BPH, advancements in energy and technology have led to the emergence of various innovative transurethral surgical therapies, such as laser procedures, bipolar enucleation, vaporization, and Aquablation®. Consequently, there is now a more diverse approach to BPH treatment [2].

Prostate enucleation is a commonly used technique that has been evolved into an endoscopic surgery, and considered a turning point in the surgical treatment of BPH [9]. It is now evident that other point sources of energy could potentially accomplish the same goal of enucleation if they are available safely [3].

The ThuLEP technique was initially introduced by Bach et al. And demonstrated immediate functional outcome with minimal complications [15]. When compared to HoLEP, ThuLEP was associated with a shorter duration of surgery, decreased decline in hemoglobin levels, and a lower occurrence of mild complications. Additionally, it significantly enhances IPSS and QoL scores in the short term (1 month), although this improvement is not sustained in the long term (12 months) [16].

Considering that BPEP exhibits comparable efficacy and safety across varying prostate sizes and has the potential to enhance urinary parameters such as IPSS score, maximum urinary flow, and post-void residual volume, it can be regarded as a procedure independent of prostate size [17]. Additionally, there are no appreciable differences between BPEP and HoLEP in terms of functional outcomes or complications [18]. In situations where laser facilities are not easily accessible, employing plasma kinetic energy for enucleation serves as a cost-effective alternative, yielding comparable outcomes to laser-based enucleation [19].

The learning curve for ThuLEP and BPEP appears to be less challenging when closely mentored, in contrast to HoLEP [20,21].

Despite a growing amount of data reported in the literature, there is a limited number of studies that have directly compared ThuLEP and BPEP for BPH management. This study aims to conduct a comparative analysis of both techniques concerning perioperative data and postoperative follow-up.

The mean enucleation time and mean total operative time were notably greater in BPEP group (75.22 ± 10.55 & 117.22 ± 17.76), respectively, than ThuLEP group (67 ± 12.18 & 90.5 ± 18.29) (*p* = 0.037 & <0.001 respectively). This may be explained by better visualization due to better hemostatic effect of thulium than plasma kinetic energy. Also, the continuous emission of the thulium laser enables precise incisions in the correct plane with minimal escahring effect being using a low power setting (60w) during cutting. Habib and his colleagues, Neill and co-authors both indicated that the duration of the PKEP procedure was lengthier than that of HoLEP [3,22], another explanations for longer operative time with BPEP was the vaporization bubbles that frequently made the tissue planes difficult to see [3].

Results showed that hemoglobin drop and intraoperative blood transfusion were more in BPEP group compared to ThuLEP group with no statistically significant difference between both groups (*p* = 0.058 & *p* = 0.546), respectively. Two patients in BPEP and one patient in ThuLEP group received intraoperative blood transfusion due to low Hb. Level (<11.5 g./dl) before surgery. This may explain the shorter catheter period and short hospital stay in ThuLEP group compared to BPEP group which may be due to better hemostasis and lower hemoglobin drop.

Previous studies [11,23,24] reported that ThuLEP was associated with diminished blood loss and a decreased requirement for blood transfusion. This can be ascribed to the vaporization characteristics of the thulium laser, which exhibits a more effective hemostatic effect compared to plasma kinetic energy. Feng and his colleagues had demonstrated that operative time was the same in both groups [9].

There was no difference in the weight of resected tissue between both groups which may be explained by using the same surgical techniques which agree with previous studies [9,22,25].

As regard postoperative functional outcomes, the curable effect of BPEP and ThuLEP was comparable with no statistically significant difference between both groups as regard IPSS, Qmax, PVRU and IIEF-5 at 1-, 3-, 6- and 12-month follow-up postoperatively [9,10]

As regard perioperative complications, both surgical methods are safe [9,10] as there is no statistically significant difference between both groups. Capsular perforation occurred in one (1.85%) patient in BPEP group and one (1.85%) patient in ThuLEP group, minor mucosal bladder injury from morcellator occurred in 1 (1.85%) patient in BPEP group and 1 patient in ThuLEP group.

Transient SUI in three patients in group A and four patients in group B with no statistically significant difference (*p* = 0.942) as previous studies [3,9,22,25]. No statistically significant difference between both groups as regard irritative urinary symptoms lasting longer than a week that were treated with anticholinergic medications and disappeared after 1 month.

Limited number of studies directly compared thulium laser enucleation and bipolar prostate enucleation. Lang Feng and his colleagues 2016 [9] proved that both BPEP and ThuLEP are effective and safe methods for treating BPH. ThuLEP offered a shorter catheter stay and a lower risk of bleeding as compared to BPEP, However, mean prostate size in this study was 69.02 mL in the ThuLEP group and 67.05 in the BPEP group compared to our study. Chen YT et al. 2022 [10] in their prospective analysis of 111 patients concluded that in terms of IPSS, quality of life, maximal flow rate, ThuLEP and BPEP were similar. In addition, ThuLEP outperformed BPEP as regard reduced blood loss. But also, with a smaller mean preoperative prostate size of 51.3 ± 20.9 for BPEP and 49.9 ± 16.1 for ThuLEP.

The present study has limitations due to its relatively small sample size and short-term follow-up. Additionally, it lacks an analysis of costs and an assessment of quality-of-life scores. Larger-scale studies with a more extended follow-up period are required to address these limitations.

## Conclusion

ThuLEP shows better perioperative parameters in comparison to BPEP. Nevertheless, there are no notable differences in functional results and complications between the two techniques. Large-scale studies with extended follow-up periods are essential to comprehensively compare their overall outcomes.
